# How do socioeconomic status relate to social relationships among adolescents: a school-based study in East China

**DOI:** 10.1186/s12887-020-02175-w

**Published:** 2020-06-03

**Authors:** Jing Li, Jing Wang, Jia-yu Li, Sheng Qian, Rui-xia Jia, Ying-quan Wang, Jing-hong Liang, Yong Xu

**Affiliations:** 1 Department of Child and Adolescent Health, School of Public Health, Medical College of Soochow University, No.199 Ren Ai Road, Suzhou, Jiangsu 215123 People’s Republic of China; 2 Business School, Xi’an International Studies University, Xi’an, 710128 People’s Republic of China; 3 Department of Social medicine, School of Public Health, Soochow University, Suzhou, Jiangsu 215123 People’s Republic of China

**Keywords:** Socioeconomic status, Parent-child relationship, Peer relationship, Student-teacher relationship, Adolescents

## Abstract

**Background:**

A great number of studies have concentrated on the influence of socioeconomic status with health outcomes, but little on how socioeconomic status affects social relationship in adolescents’ families, peers and schools. This study aimed to clarify more detailed information on the connection between social relationships and different dimensions of socioeconomic status.

**Methods:**

A school-based cross-sectional study was performed by 13–18 adolescents enrolled in East China from September, 2018 to May, 2019, which recruited 6902 students from junior and senior high schools and used the stratified random sampling method. Parent-child relationship (cohesion, expressiveness, conflict), peer relationship (interpersonal relationship, communication and interaction, social emotion) and student-teacher relationship (intimacy, support, satisfaction, conflict) were investigated. Besides, objective socioeconomic status (parental education and occupation, assessed by the adolescent) and subjective socioeconomic status (self-evaluation of family social class) were measured. More detailed information was used to clarify the link between social relationships and different dimensions of socioeconomic status.

**Results:**

All five indicators of socioeconomic status were slightly positively correlated with the quality of social relationships (r ranged from 0.036 to 0.189, all *p* < 0.001), except that maternal education was not correlated with the conflict dimension of parent-child relationship. Standardized regression coefficients indicated that paternal education (β = 0.08) and occupation (β = 0.07) were the predictors of parent-child relationship. And peer relationship model revealed that the corresponding effect size was slightly stronger for subjective socioeconomic status (β = 0.10), whereas the maternal education had a slightly stronger correlation with student-teacher relationship (β = 0.07) relative to other indicators.

**Conclusions:**

Adolescents with lower socioeconomic status had poorer social relationships compared to those with higher socioeconomic status. These findings have important public health implications for health policy makers to make sound decisions on resources allocation and services planning in improving adolescents’ social relationships and promoting health outcomes.

## Background

Socioeconomic status (SES), as one of the most significant social determinants of health, has attracted increasing attention around the globe. Many studies have suggested that education, income and occupation of objective SES indicators have profound impacts on everyone’s health [1, 2]. Subjective SES is also called as subjective status, perceived social position [3], and subjective social status [4]. It is defined as the faith of an individual about his or her position in the socioeconomic structure [5]. Some researchers demonstrate that subjective SES is more accurate in capturing the more subtle aspects of social status, and providing more information than objective SES indicators, it also has a greater impact on health [6].

The effects of SES on health change over the life course [7]. The importance of socioeconomic factors for infants’ [8] and adults’ [9] health have been widely demonstrated to be inverse, and graded correlated. However, there is little evidence that adolescents’ health impacted by SES may be consistent [10]. For example, some studies showed that there was an inverse gradient among SES, global health indicators, acute conditions, and health behaviors [11, 12]. Meanwhile, some results indicated that SES had no gradient among non-fatal injury, acute illness, mental health, and self-rated health [13, 14].

Additionally, it is obvious that the ecological environment and relationships of adolescents’ development have changed significantly as individuals transfer from childhood to adolescence, however, family, peers and school are still the most important and direct social context at the microsystem level [15]. A good parent-child relationship is a vital protective factor for adolescents [16]. Peer relationship refers to the relationship which was formed by common activities and mutual contacts between adolescents of the same or similar age. Healthy peer relationship is critical to the positive development of adolescents’ cognitive, emotional, social skills, and scholastic adaptation, which support them in normative transitions of development [17] and buffer them against the impact of burdensome circumstances in other areas of life [18]. Student-teacher relationship is a basic interpersonal connection developed by prolonged interaction between students and teachers, which reflects their psychological state of seeking satisfaction through emotional, cognitive and behavioral communication [19]. Positive student-teacher relationship is critical to the positive development of adolescents. It provides security, safety, and protection that necessary for students’ full participation in social activities [20], and supports them in adjusting to school life, improving their social skills and promoting academic achievement [21]. Drawing from these perspectives, social relationship, including parent-child relationship, peer relationship and teacher-student relationship, is an important factor influencing many health outcomes of adolescents, such as health behaviors, mental health, physical health and death risk [22].

Whereas a lot of researches have focused on adolescents’ health outcomes in terms of socioeconomic status or social relationships, few of them know about social relationships from the perspective of socioeconomic status. The current study investigated the family, peer and student-teacher relationships of adolescents in East China to clarify more detailed information on the connection between these relationships with different dimensions of socioeconomic status. We sought to figure out whether subjective SES and objective SES differed in the strength of adolescents’ social relationships with different dimensions.

## Methods

### Participants and procedure

**Fig. 1:**
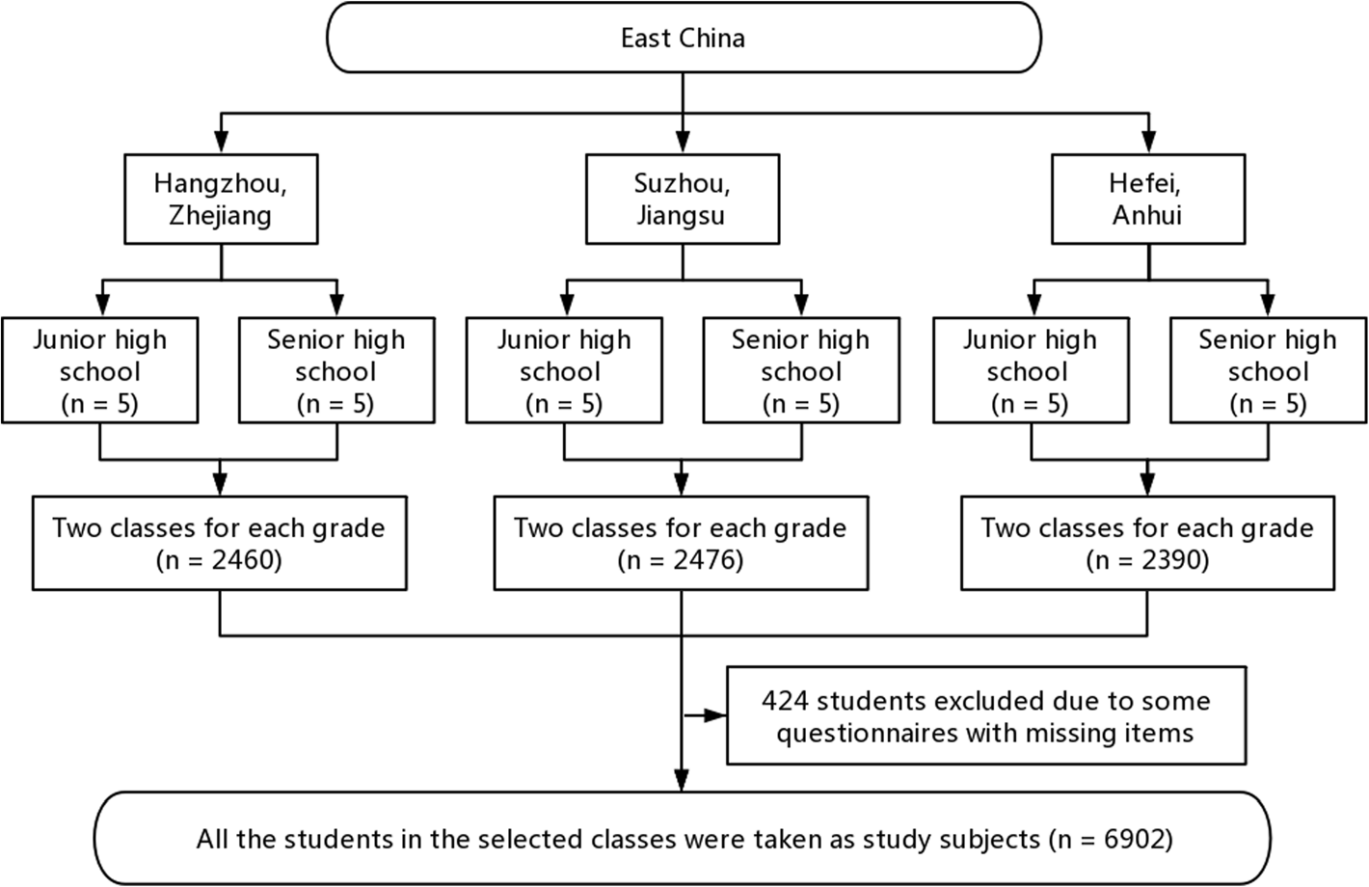
Flowchart of sampling and subject recruitment

From September, 2018 to May, 2019, a school-based cross-sectional study was performed by adolescents enrolled in East China. There were 6902 students came from middle or high school, including 3355 males and 3547 females. A stratified cluster random sampling method was performed through four stages of selection **(**Fig. 1**)**. In the first stage, three administrative regions of Hangzhou, Suzhou and Hefei were selected from Zhejiang, Jiangsu and Anhui provinces, respectively. Next, five junior high schools and five senior high schools were selected from each of the three regions. Thirdly, each grade was considered as a single sampling stratum, and two classes were randomly selected from each grade of the 30 schools. Finally, all the students in the selected classes were taken as study subjects. The inclusion criteria for the study participants were as follows: (a) aged 13 to 18, (b) all of them lived in Suzhou or Hefei or Hangzhou with their parents for more than 6 months prior to the start of the study, and (c) voluntary participation. Participants with the following characteristics were excluded: (a) psychological or mental health issues, and (b) absence during the survey. The investigation was conducted during the school year, avoiding the school examination period. In cooperating schools, teachers were required to inform students about the study and to ensure privacy. Each of these steps were explained in detail by the investigator. All participants filled out the questionnaires anonymously after knowing the purpose and methods of the survey. In addition, the study was investigated in the absence of teachers.

Previous study has shown that 5.2% of middle school students in China have a variety of significant mental health problems [23]. So we estimated the minimum sample size needed for this study based on this prevalence. The calculation formula was as follows:

$$ n={\left\{\frac{57.3{Z}_{\alpha /2}}{\arcsin \Big[\varepsilon P/\sqrt{P\left(1-P\right)}}\right\}}^2 $$

In the present study, p was the prevalence of mental health problems (p = 0.052), ɛ was the error tolerance (ɛ = 0.1), alpha (α) was the significance level (α = 0.05, Z_α/2_ = 1.96 accordingly), the estimated minimum sample size was 6920. We added an additional 10% to the minimum sample size, taking into account the non-response rate or absence, and targeted at 7612 subjects. A total of 7326 students participated in the survey. In the end, only 6902 valid surveys were retained after excluding questionnaires with missing items.

### Measurement

#### Sociodemographic variables

The social and demographic characteristics of the participants were recorded in the questionnaires, including the age, grade and household registration of the respondents. Information from student interviews was used to assess the family structure based on the person that adolescents were living with. The options available were father and mother, father and stepmother/mother and stepfather, father only/mother only, and some other legal guardian. For the analyses, we created a dichotomous variable: intact family (natural parents)/non-intact family (stepparent or single parent or other legal guardian). The variable of family size was also obtained through student interviews, which can be divided into two categories (non-only child/only child).

#### Socioeconomic status

The SES of families was used to reflect the SES of adolescents, which included both subjective SES and objective SES (education and occupation of parents) [24]. All of indicators were assessed by students, which saved the cost and time of data collection [25].

As for education, adolescents were required to indicate the highest level of education completed by each parent, and we provides the following options: uneducated, preschool, primary school, junior high school, senior high school/ vocational school, college/university, graduate school. Then it was recoded as low (≤ ISCED 0, i.e. uneducated, preschool), medium (ISCED 1–2, i.e. primary school, junior high school), and high (ISCED 3–8, i.e. senior high school/ vocational school, college/university, graduate school) according to the International Standard Classification of Education (ISCED) [26].

Students were also asked to answer where their parents worked and what job they did. When adolescents were allowed to answer these open-ended questions, the proportion of non-response or unclassifiable answers associated with this measurement showed a significant decrease [27]. Afterwards, based on the International Standard Classification of Occupations (ISCO-08) [28], each parent’s occupation status was recalculated. The occupational status was divided into three categories based on whether the parent had a job, which type of work and what skill level as: low prestige (unemployed), medium prestige (temporary worker, clerks, service and sales, skilled workers, craft and related, plant/machine operators, elementary occupations, and armed forces), and high prestige (technicians, professionals and managers).

Subjective social status required adolescents to evaluate the socioeconomic status of their families [29]. It was consisted of a 5-point ladder scale: 1 (the most affluent) - 5 (the most disadvantaged). For the convenience of analysis, we divided the answers into the following three categories: lower class (1–2, below average), middle class (3, average), and upper class (4–5, above average).

#### Social relationships

Parent-child relationship: The Family Environment Scale (FES) was a self-report tool used to evaluate the social environment of the family [30]. It has been shown to have good reliability and validity in samples of Chinese adolescents [31]. It contained 10 sub-scales and involved three broad domains: social relationship within the family, personal growth or goal orientation, and system maintenance. In this study, we specifically selected sub-scales of cohesion, expressiveness, and conflict to evaluate the quality of parent-child relationship. Moreover, this domain was consisted of 27 true or false items, and each sub-scale contain 9 items. The conflict was scored reversed so that three sub-scales were scored in the same direction [30]. Total score of this domain was 0–27 points, and higher score indicated more harmonious of parent-child relationship. The Cronbach’s alpha value for this sample was 0.71.

Peer relationship: The students were asked to describe the quality of their relationship with peers using the Peer Relationship Scale developed by Wei Yunhua, a Chinese scholar [32]. The reliability and validity of the Peer Relationship Scale have been confirmed in previous studies on adolescents [33]. This questionnaire was consisted of 20 questions, involving three domains: interpersonal relationship, communication, interaction, and social emotion. Each item was assessed on a five-point Likert scale from definitely does not apply (1 point) to definitely apply (5 points). Total score of this scale was 20–100 points with higher scores reflecting a better-quality relationship between the adolescents and peers. The total measurement yielded an internal consistency (Cronbach’s α) of 0.90 in the present study.

Student-teacher relationship: The students were asked to describe the quality of their relationship with teachers using the Chinese version of the Student-Teacher Relationship Scale, which was originally developed by Pianta [34] and revised by Zhiyong Qu [35]. The Chinese version of this scale has been widely used in middle school students in China [36]. Students rated their perceptions of their relationship with the teachers on 23 items, which assessed the level of support, satisfaction, intimacy, and conflict in their relationship. Each item used a 5-point Likert scale (definitely does not apply to definitely applies) and total score was created from the sum of the 23 items with higher scores reflecting a better-quality relationship between the adolescents and teachers. The subscale of conflict was scored reversed, so a positive relationship characterized by trust, warmth, and low conflict was represented by high scores. The study was reliable and adequate with Cronbach’s alpha values of 0.83 for total scale.

### Statistical analysis

The statistical analysis was performed by using the software IBM SPSS version 23. A *p*-value of < 0.05 was considered statistically significant (two-tailed). Descriptive statistics of the presentation of demographic data and socioeconomic status were performed and presented as appropriate frequencies and proportions. The links between SES indicators and scores on different dimensions of social relationships were examined by using Spearman correlations analyze. The correlation of r = 0.1 was interpreted as small, *r* = 0.3 was medium, *r* = 0.5 was large [37]. Furthermore, the correlation of SES with social relationships were investigated by using hierarchical multiple regression. Adjusted *R*^*2*^ statistics were used to evaluate the overall fit of the model [38]. Standardized regression coefficients were computed to evaluate the relative contributions of these variables to variations in social relationships.

## Results

In this cross-sectional study, there were more junior high school students (61.0%) than senior high school students (39.0%). The mean ages (SD) of junior and senior high school students were 13.9 (0.8) and 16.9 (0.8) years, respectively. There were 41.7% of adolescents from urban areas and 58.3% from rural areas. Most of the students in the sample came from intact families (83.6, and 85.9% for junior and senior high school students, respectively). With regarded to subject SES, a larger number of students thought they belonged to middle class, both in junior high school students (68.9%) and senior high school students (71.0%). Nearly two thirds of adolescents reported that their parents had a medium level of education, while nearly half reported that their parents had a medium prestige occupation. The average score (SD) of family, peer and student-teacher relationship were 18.18 (4.20), 64.90 (13.38) and 76.00 (12.43), respectively. (Table 1).

**Table 1:** Demographic characteristics of the adolescents in East China (n = 6902)

Characteristics	Junior high school (*n* = 4208)	Senior high school (*n* = 2694)	*X*^*2*^	*p*
**Gender**				
Boys	2029 (48.2)	1326 (49.2)	0.661	0.416
Girls	2179 (51.8)	1368 (50.8)		
**Household registration**				
Urban	1886 (44.8)	992 (36.8)	43.205	< 0.001
Rural	2322 (55.2)	1702 (63.2)		
**Family structure**				
Intact family	3516 (83.6)	2315 (85.9)	7.076	0.008
Non-intact family	692 (16.4)	379 (14.1)		
**Family size**				
Non-only child	1997 (47.5)	1535 (57.0)	59.592	< 0.001
Only child	2211 (52.5)	1159 (43.0)		
**Subjective SES**				
Lower class	605 (14.4)	517 (19.2)	81.416	< 0.001
Middle class	2899 (68.9)	1914 (71.0)		
Upper class	704 (16.7)	263 (9.8)		
**Paternal education**				
Low	67 (1.5)	75 (2.8)	17.935	< 0.001
Medium	2607 (62.0)	1728 (64.1)		
High	1534 (36.5)	891 (33.1)		
**Maternal education**				
Low	243 (5.7)	205 (7.6)	18.848	< 0.001
Medium	2742 (65.2)	1812 (67.3)		
High	1223 (29.1)	677 (25.1)		
**Paternal occupation**				
Low	68 (1.6)	46 (1.7)	3.986	0.136
Medium	2299 (54.6)	1535 (57.0)		
High	1841 (43.8)	1113 (41.3)		
**Maternal occupation**				
Low	612 (14.5)	359 (13.3)	20.774	< 0.001
Medium	2061 (49.0)	1470 (54.6)		
High	1535 (36.5)	865 (32.1)		

Moreover, all five indicators of SES were weak positively correlated with the quality of social relations (r ranged from 0.036 to 0.189, all *p* < 0.001), except that maternal education level was not correlated with the conflict dimension of parent-child relationship. To be specific, adolescents with lower subjective SES, lower education level and lower prestige occupation status of those parents reported lower quality child-parent, peer, student-teacher relationship than adolescents with higher family SES. (Table 2).

**Table 2:** Spearman’s correlation coefficients between SES indicators and different domains of social relationships among adolescents

SES	Parent-child relationship			Peer relationship			Student-teacher relationship			
	Cohesion	Expressiveness	Conflict	Social emotion	Communicative interaction	Interpersonal concordance	Intimacy	Support	Satisfaction	Conflict
Subjective SES	0.037**	0.054**	0.048**	0.120**	0.152**	0.168**	0.087**	0.084**	0.092**	0.036**
Paternal education level	0.110**	0.080**	0.049**	0.158**	0.153**	0.189**	0.119**	0.094**	0.082**	0.059**
Maternal education level	0.062**	0.060**	0.023	0.165**	0.156**	0.188**	0.134**	0.087**	0.079**	0.089**
Paternal occupation status	0.112**	0.067**	0.061**	0.142**	0.137**	0.165**	0.095**	0.083**	0.063**	0.049**
Maternal occupation status	0.056**	0.041**	0.049**	0.091**	0.083**	0.117**	0.090**	0.056**	0.052**	0.047**

** Correlations were significant at the 0.01 level (2-tailed)

Table 3 presented the results of hierarchical multiple linear regression. The Model 1 of child-parent, peer, and student-teacher relationships (adjusted *R*^*2*^ was 0.025, 0.041, 0.057, respectively) indicated that girls, urban household registration, intact family were significantly associated with higher quality social relationships. Additionally, non-only child had higher quality of parent-child relationship, and older adolescents had lower quality of peer and student-teacher relationship.

**Table 3:** Hierarchical multiple regression of social relationships with SES variables among adolescents

Variables	Model 1				Model 2			
	B	95% (CI)	*p*	β	B	95% (CI)	*p*	β
**Parent-child relationship**								
Age (Years)	−0.02	− 0.08, 0.04	0.52	−0.01	− 0.01	− 0.07, 0.05	0.70	− 0.01
Gender (Boy vs. Girls)	0.73	0.53, 0.92	< 0.001	0.09	0.71	0.51, 0.91	< 0.001	0.08
Household registration (Urban vs. Rural)	−0.23	− 0.44, − 0.03	0.03	− 0.03	0.07	− 0.15, 0.30	0.53	0.01
Family structure (Intact vs. Non-intact)	−1.38	−1.65, −1.11	< 0.001	− 0.12	− 1.28	− 1.55, − 1.01	< 0.001	− 0.11
Family size (Non-only child vs. only child)	− 0.52	− 0.72, − 0.31	< 0.001	− 0.06	− 0.49	− 0.70, − 0.28	< 0.001	−0.06
Subjective SES					0.18	−0.01, 0.37	0.06	0.02
Paternal education level					0.65	0.42, 0.89	< 0.001	0.08
Maternal education level					−0.20	−0.42, 0.03	0.09	−0.03
Paternal occupation status					0.54	0.32, 0.77	< 0.001	0.07
Maternal occupation status					−0.10	−0.27, 0.07	0.25	−0.02
Adjusted *R*^*2*^	0.025				0.035			
∆*R*^*2*^	0.026				0.011			
**Peer relationship**								
Age (Years)	−0.42	−0.6, −0.23	< 0.001	−0.05	− 0.29	−0.48, − 0.11	< 0.001	−0.04
Gender (Boy vs. Girls)	1.95	1.33, 2.58	< 0.001	0.07	1.71	1.10, 2.33	< 0.001	0.06
Household registration (Urban vs. Rural)	−3.40	−4.05, −2.75	< 0.001	−0.13	−0.94	−1.64, − 0.24	0.01	− 0.04
Family structure (Intact vs. Non-intact)	−4.43	−5.29, − 3.57	< 0.001	− 0.12	− 3.73	−4.58, − 2.89	< 0.001	−0.10
Family size (Non-only child vs. only child)	−0.46	− 1.11, 0.19	0.17	−0.02	0.09	−0.56, 0.74	0.79	0.003
Subjective SES					2.45	1.86, 3.03	< 0.001	0.10
Paternal education level					2.04	1.30, 2.77	< 0.001	0.08
Maternal education level					1.92	1.21, 2.62	< 0.001	0.08
Paternal occupation status					2.07	1.38, 2.77	< 0.001	0.08
Maternal occupation status					−0.12	−0.65, 0.41	0.67	−0.01
Adjusted *R*^*2*^	0.041				0.081			
∆*R*^*2*^	0.041				0.041			
**Student-teacher relationship**								
Age (Years)	−1.17	−1.34, −1.00	< 0.001	−0.16	−1.11	−1.28, − 0.93	< 0.001	−0.15
Gender (Boy vs. Girls)	2.29	1.71, 2.87	< 0.001	0.09	2.12	1.54, 2.7	< 0.001	0.09
Household registration (Urban vs. Rural)	−1.44	−2.04, −0.84	< 0.001	− 0.06	0.06	− 0.6, 0.71	0.87	0.002
Family structure (Intact vs. Non-intact)	−4.80	−5.58, −4.01	< 0.001	−0.14	−4.37	−5.16, −3.58	< 0.001	− 0.13
Family size (Non-only child vs. only child)	−0.05	− 0.65, 0.55	0.88	− 0.002	0.36	− 0.25, 0.96	0.25	0.01
Subjective SES					0.90	0.36, 1.44	< 0.001	0.04
Paternal education level					1.15	0.47, 1.84	< 0.001	0.05
Maternal education level					1.52	0.86, 2.18	< 0.001	0.07
Paternal occupation status					1.13	0.48, 1.77	< 0.001	0.05
Maternal occupation status					0.20	−0.29, 0.7	0.42	0.01
Adjusted *R*^*2*^	0.057				0.072			
∆*R*^*2*^	0.057				0.016			

Model 2 included the five SES indicators and the control variables (i.e., age, gender, household registration, family structure, family size). In parent-child relationship model (adjusted *R*^*2*^ = 0.035), the two single indicators of SES were statistically significant. Standardized regression coefficients indicated paternal education level (β = 0.08) and paternal occupation status (β = 0.07) were the predictors of parent-child relationship. Peer relationship model (adjusted *R*^*2*^ = 0.081) revealed that higher subjective SES, higher level of parents’ education, and higher prestige paternal occupation status meant the adolescents had a better relationship with peers. The corresponding effect sizes were small and slightly stronger for subjective SES (β = 0.10) than other three SES indicators (all β = 0.08). In student-teacher relationship model (adjusted *R*^*2*^ = 0.072), it was interesting that subjective SES, parents’ education level and fathers’ occupation status were still significant, but the maternal education level only had a slightly stronger correlation (β = 0.07) than other indicators.

## Discussion

Greater attention has been paid to determinants of the health and well-being of adolescents, including SES and the social environment of family and school [39]. In this school-based cross-sectional study, we found that adolescents with low SES had lower quality of social relationships. We also noticed that subjective SES was a relatively stronger predictor for peer relationship than objective SES, while objective SES could better predict parent-child relationship and student-teacher relationship among adolescents. These data have provided valuable information for improving social relationships and promoting health among adolescents.

In this study, adolescents’ self-reported subjective SES and objective SES indicators (parental occupation and education) were used to observe their social relationship. And as expected, we found that adolescents with higher subjective SES had better relationship with their peers. These results extend previous studies that positive peer and student-teacher relationships are more likely to be established by adolescents with higher subjective SES [40]. There are strong relationships between subjective SES and health outcomes, which can be explained by several reasons [6]. First of all, subjective SES may reflect a relative social status of one person in social class, moderate the relationship between income inequality and population health, rather than demonstrate the absolute status of one [41]. Secondly, subjective SES may be a more accurate measurement of social status that taken past and future prospects into account, and making more nuanced judgments for objective indicators, which can represent the cognitive average of various socioeconomic status indicators [5]. Lastly, a reciprocal relationship may exist between subjective SES and health [3].

On the other hand, our analysis also provided evidence that subjective SES was a relatively stronger predictor of peer relationship than objective SES. Adolescents are in a special period when they may develop a sense of social status during the transition between childhood dominated by family status and adulthood dominated by self-determination. The ecological environment constructed in the process of human development is a dynamic system. So adolescents are more involved in the social environment outside the family, especially the interaction with peers. And a number of studies confirmed that the content of friendships changes as children enter adolescence [42]. At the early stage of adolescence, individuals have a superficial understanding of friendship and pay attention to common activities. In the middle of puberty, there are more emphasis on mutual emotional dependence between peers, especially on loyalty, trustworthiness and respect. Older adolescents generally believe that friends need to understand and support each other, which involves deep psychological consistency in personality. Previous studies have shown that subjective SES in adolescence may reflect the influence of objective SES and modern consumer culture (emphasis on property, brand and/or appearance) [43]. Additionally, adolescents with lower subjective SES are more likely to experience higher stress from different areas including adverse social relationships [44]. Adolescents with higher SES may have more sense of superiority and self-identity than other peers. They are also more attractive and mature, which makes them easier to be accepted by peers in interpersonal communication, thus satisfying their needs for peers at different times [45].

Focusing on objective SES, our result revealed that the education of father and mother was the main predictor of parent-child relationship and student-teacher relationship, respectively. This is consistent with a number of studies that have shown a relevance between parental higher education and various positive health outcomes [46]. Furthermore, the most widely used measure standard of SES among adolescents is parental education level [47]. The advantage of the index is that it is easy to measure and it can strongly predict employment and income levels [48], which is considered to be the most effective predictor of adolescent health [2].

Knowledge, social status and available resources could be all reflected in education. And the higher of parents’ education, the more conducive to develop adolescents’ interpersonal relationships [49]. Family is the first and most important factor that influences children’s socialization. As important persons in children’s life, parents have close emotional contacts with children, and the most frequent participation and management in their children’s social life. Therefore, parents have very important influences on all aspects of children’s life, including interpersonal relationship [50]. It is noticed that some behavioral influences posed on children’s development, such as lifestyle choices, parenting styles, knowledge and skills are linked to parental education [51]. Highly educated parents have a better understanding of child development and are able to choose a more appropriate parenting type and practice [52]. Parents play different roles in the family, under the influence of Chinese traditional social culture, mothers often spend a lot of time in the family and have more responsibility for taking care and educating adolescents. While fathers pay more attention to the development of the career and provide solid economic foundation for the family, who tend to lack of effective and in-depth communication with adolescents [53]. In addition, during the transition from childhood to adulthood, adolescents become more self-awareness and are more involved in social relations outside the family along with the rapid, novel and unexpected changes occurred in the physical, psychological, and social development [15]. Therefore, in the process of raising adolescents by parents, mothers with high education level can better guide adolescents to establish positive social relationship, including student-teacher relations [54]. Highly educated fathers are more likely to maintain a good parent-child relationship, for they are more aware of the problems and adverse effects, and can provide measures to improve the situation [55].

This school-based study may lead to a deeper and more diverse understanding of the relationship between adolescent social relationships and socioeconomic factors. Our research focuses on the multi-dimensional family socioeconomic status in attempt to capture the social status of family differentially and extensively. Additionally, all measurements were self-reported, except that students were asked to fill out questionnaires anonymously without the presence of teachers to reduce social desirability bias. While there are some limitations in the current study. First of all, our analysis was based on cross-sectional data, which limited our ability to confidently infer the direction of causality. Secondly, income as one of the main indicators of socioeconomic status was not included in this study, because it was generally considered as sensitive information in China and the authenticity of the obtained data couldn’t be guaranteed. But researchers are encouraged to include this indicator, as it is relatively independent influence [51]. Thirdly, the study only analyzed the socioeconomic status of adolescents’ family, while ignoring their school SES. Finally, the representativeness of the results may be limited, as only middle school students’ data were collected in this study and the same number of schools were selected from three cities without the estimated weight adjustment. Nevertheless, it had substantive, practical and methodological implications, which increased small body of work on socioeconomic status and social relations.

## Conclusion

Substantively, we found the inequity existed in social relationships of adolescents from East China. Adolescents with lower SES had poorer social relationships relative to higher SES. And subjective SES, paternal education and maternal education were the main predictors of peer relationship, parent-child relationship and student-teacher relationship, respectively. An understanding of the effects of socioeconomic on social relationships will prompt public health experts and policy makers to identify, intervene, and eventually alleviate the root causes of adolescents’ health-associated problems. As such, preventative programs and services for adolescents with low parental SES as well as low subjective SES should be provided. These programs should include social support and education to raise awareness of the problems faced by adolescents and their families and to address them so as to improve their outcomes and avoid the negative effects of low SES [56].

## Data Availability

Datasets used and analyzed during this study are available from the corresponding author on reasonable request.

## Abbreviations

SES: Socioeconomic status
FES: Family Environment Scale
ISCO: International Standard Classification of Occupations
ISCED: International Standard Classification of Education
